# Methoxymethyl (MOM) Group Nitrogen Protection of Pyrimidines Bearing C-6 Acyclic Side-Chains 

**DOI:** 10.3390/molecules16065113

**Published:** 2011-06-20

**Authors:** Tatjana Gazivoda Kraljević, Martina Petrović, Svjetlana Krištafor, Damjan Makuc, Janez Plavec, Tobias L. Ross, Simon M. Ametamey, Silvana Raić-Malić

**Affiliations:** 1 Department of Organic Chemistry, Faculty of Chemical Engineering and Technology, University of Zagreb, Marulićev trg 20, HR-10000 Zagreb, Croatia; 2 Slovenian NMR centre, National Institute of Chemistry, Hajdrihova 19, SI-1000 Ljubljana, Slovenia; 3 EN-FIST Centre of Exellence, Dunajska 156, SI-1000 Ljubljana, Slovenia; 4 Faculty of Chemistry and Chemical Technology, University of Ljubljana, Aškerčeva cesta 5, SI-1000 Ljubljana, Slovenia; 5 Radiopharmaceutical Chemistry, Institute of Nuclear Chemistry, Johannes Gutenberg-Universität, Fritz-Strassmann Weg 2, 55128 Mainz, Germany; 6 Center for Radiopharmaceutical Sciences, ETH Zurich (Swiss Federal Institute of Technology), Wolfgang-Pauli Strasse 10, CH-8093 Zurich, Switzerland

**Keywords:** *N*-methoxymethyl protecting group, C-6 isobutyl pyrimidine derivatives, *N*-1 and *N*-3 regioisomers, NOE experiments, positron emission tomography (PET)

## Abstract

Novel *N*-methoxymethylated (MOM) pyrimidine (**4**−**13**) and pyrimidine-2,4-diones (**15**−**17**) nucleoside mimetics in which an isobutyl side-chain is attached at the C-6 position of the pyrimidine moiety were synthesized. Synthetic methods *via* *O*-persilylated or *N*-anionic uracil derivatives have been evaluated for the synthesis of *N*-1- and/or *N*-3-MOM pyrimidine derivatives with C-6 acyclic side-chains. A synthetic approach using an activated *N*-anionic pyrimidine derivative afforded the desired *N,N*-1,3-diMOM and *N*-1-MOM pyrimidines **4 **and **5** in good yield. Introduction of fluorine into the side-chain was performed with DAST as the fluorinating reagent to give a *N,N*-1,3-diMOM pyrimidine **13 **with a 1-fluoro-3-hydroxyisobutyl moiety at C-6. Conformational study of the monotritylated *N*-1-MOM pyrimidine **12** by the use of the NOE experiments revealed the predominant conformation of the compound to be one where the hydroxymethyl group in the C-6 side-chain is close to the *N*-1-MOM moiety, while the OMTr is in proximity to the CH_3_-5 group. Contrary to this no NOE enhancements between the *N*-1-MOM group and hydroxymethyl or fluoromethyl protons in **13** were observed, which suggested a nonrestricted rotation along the C-6 side-chain. Fluorinated *N,N-*1,3-diMOM pyrimidine **13** emerged as a model compound for development of tracer molecules for non-invasive imaging of gene expression using positron emission tomography (PET).

## 1. Introduction

The pyrimidine moiety is a widespread heterocyclic unit which is found in several biologically active natural products, as well as synthetic pharmacophores with biological activities that show considerable therapeutic potential [1,2,3,4,5]. The structural diversity and biological importance of pyrimidines have made them attractive targets for synthesis over the years. For this reason numerous analogues and derivatives of pyrimidines have been synthesized and developed as pharmacologically active compounds or drugs [6,7]. 

Many *N*-substituted uracil derivatives have exhibited extremely diverse physiological activity [8]. It was shown that *N*-1 and/or *N*-3-alkylated pyrimidine derivatives had a wide range of antiviral activity [9,10,11,12,13,14]. A large number of thymidine analogues and acyclic guanosine derivatives showed antiviral activities against herpes simplex virus type 1 (HSV-1) and 2 (HSV-2) [15]. The antiviral activity of these compounds is due to their selective and efficient *in vivo* activation through monophosphorylation by the viral enzyme [16,17]. The monophosphates are converted to diphosphates, and then to the corresponding triphosphates by cellular enzymes. The triphosphates prevent viral replication by inhibition of the viral DNA polymerase [18]. The molecular basis of the therapy, which uses viral thymidine kinase (TK), is the difference in substrate specificity between the herpes viral TK and the human cellular isoenzyme. Therefore, HSV-1 TK in combination with nucleoside analogue as fraudulent substrates can be used as suicide enzymes in gene therapy of cancer [19,20,21]. Furthermore, these compounds labelled with positron-emitting radioisotopes can be used as *in situ* reporter probes to allow non-invasive imaging of HSV-1 TK gene expression using positron emission tomography (PET) [22,23,24,25]. The pronounced biological activities exhibited by C-6 substituted pyrimidine derivatives provide a good rationale for further exploration of the chemistry and biological activities of these compounds [26,27,28,29,30]. Thus, we have synthesized nucleoside mimetics in which acyclic sugar moiety is attached at the C-6 position. Some C-6 fluoroalkylated pyrimidines exhibited pronounced cytostatic activities [31,32,33], while thymines with 6-(2,3-dihydroxypropyl) and 6-(1,3-dihydroxyisobutyl) side-chains have been developed as tracer molecules for monitoring of HSV-1 TK expression by means of PET [34,35,36]. Our investigations were prompted by the need to develop PET imaging agents which lack the disadvantages of already existing reporter probes of HSV-1 TK, such as 9-[4-[^18^F]fluoro-3-(hydroxymethyl)butyl]guanine ([^18^F]FHBG) which shows cytotoxicity and unfavorable pharmaco-kinetics [37]. In our previous work we described the compound 6-(1,3-dihydroxyisobutyl)thymine (DHBT) and discussed its advantages over existing compounds [35]. The molecular modeling and the X-ray structure of HSV-1 TK in complex with *N*-methylated DHBT gave new insights for the design and synthesis of further C-6 substituted pyrimidine derivatives with differentiated pharmacokinetics [37]. 

In the view of the facts mentioned above and in continuation of our previous work on development of tracer molecules for non-invasive imaging of gene expression using PET, we have now prepared new *N*-methoxymethylated (MOM) C-6 acyclic pyrimidine derivatives. Thus, herein we report syntheses of novel *N*-1- and/or *N*-3-MOM pyrimidines **4**−**13** and pyrimidin-2,4-diones **15**−**17** bearing C-6 isobutyl side-chains, as well as a bicyclic pyrimido[1,6-*c*][1,3]oxazepine derivative **14**. 

## 2. Results and Discussion

### 2.1. Chemistry

**Scheme 1:**
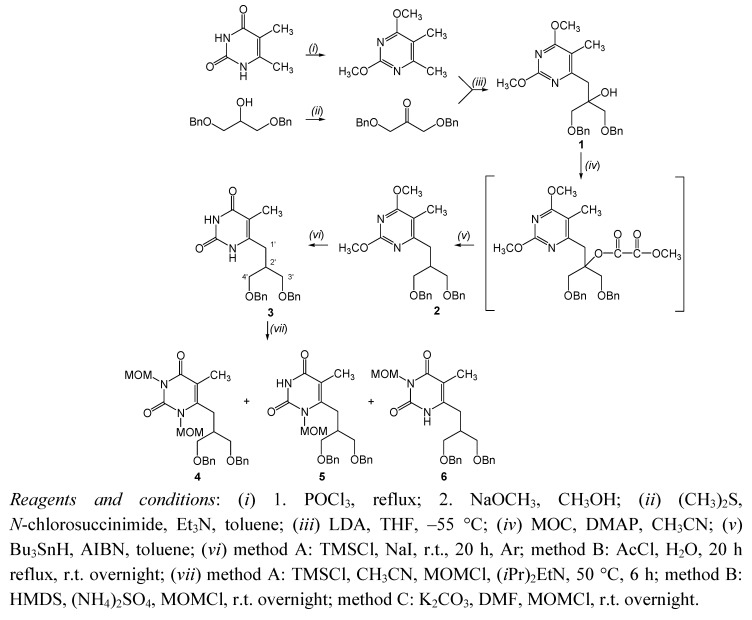
Synthetic pathway from 5,6-dimethylpyrimidine to the C-6 substituted pyrimidine derivatives **1-6**.

Synthesis of the pyrimidine scaffold **2 **was achieved according to the previously reported procedure (Scheme 1) [38]. Treatment of 2,4-dimethoxy-5,6-dimethylpyrimidine with lithium diispropylamide (LDA) in THF at −55 °C afforded the corresponding lithiated precursor, which reacted *in situ* with 1,3-dibenzyloxy-2-propanone to give 6-(1,3-dibenzyloxy-2-hydroxyisobutyl)thymine (**1**) [35]. 

The lithiation reaction has been method for introducing various functionalities into the pyrimidine moiety [39]. Reaction of **1** with methyl oxalyl chloride gave oxalate, which was submitted to Barton-McCombie deoxygenation using tributyltin hydride and 2,2'-azobis(isobutyronitrile). This is radical substitution in which ester group in oxalate is replaced by a hydride to give pyrimidine derivative containing 3-benzyloxy-2-(benzyloxymethyl)propyl side-chain (**2**). 

Hydrolysis of the 2,4-dimethoxy group in **2** was accomplished with trimethylsilyl iodide generated *in situ* from trimethylsilyl chloride and sodium iodide to afford pyrimidin-2,4-dione **3** in 18% yield (Scheme 1). Demethoxylation of **2** with acetyl chloride (AcCl) and water gave the same product **3** in improved yield (68%). To avoid intramolecular cyclization and formation of a conformationally constrained carbon-bridged pyrrolido[1,2-*c*]pyrimidine, formed by *N*-1 linkage to the acyclic moiety at C-6 position [35,38], a strategy involving *N-*methoxymethylation of **3** was applied with methoxymethyl chloride (MOMCl) as alkylating reagent. 

**Table 1:** Overview of used synthetic methods, reaction conditions and yields.

Entry	Reaction	Starting compd	Reagents and conditions	Product	Yield (%)
1	demethoxylation	**2**	TMSCl, NaI, CH_3_CN, r.t., 20 h	**3**	18.3
			AcCl, reflux, 20 h, H_2_O, r.t., overnight		67.8
2	methoxymethylation	**3**	TMSCl, CH_3_CN, reflux, 2 h, ( *i*Pr)_2_EtN, MOMCl, 50 °C, 6 h	**4**	22.4
				**5**	13.4
				**6**	11
			HMDS, (NH_4_)_2_SO_4_, reflux, 1 h, MOMCl, r.t., overnight	**4**	2.9
				**5**	21.4
			K_2_CO_3_, DMF, MOMCl, r.t., overnight	**4**	28.5
				**5**	12.1
				**6**	15.4
3	debenzylation	**4**	BCl_3_, CH_2_Cl_2_, −70 °C, 1h	**7**	33.4
				**8** and **9**	24.8
4		**5**	BCl_3_, CH_2_Cl_2_, −70 °C, 4 h	**8**	18.6
				**15**	60
			BCl_3_, CH_2_Cl_2_, −70 °C, 2 h	**8**	31.7
5	tritylation	**7**	MTrCl, DMAP, DMF, r.t., overnight	**10**	46.4
				**11**	37.5
				**12**	5.5
6		**8**		**12**	30.8
7		**15**		**16**	26.12
				**17**	9.4

As the literature precedents attest [40,41,42,43,44] there are only a few reports on the MOM protection of nitrogen in a pyrimidine ring, and no report of *N*-MOM derivatives with C-6 isobutyl side-chains. Generally, *N-*methoxymethylation is accomplished by the substitution of MOMCl with activated *O*-persilylated or *N*-anionic uracil derivatives. In our method A chlorotrimethylsilane (TMSCl) was applied as a silylating agent and the reaction was conducted in CH_3_CN in the presence of diisopropylethylamine [(*i*Pr)_2_EtN]. This reaction was performed under strictly anhydrous conditions due to the fact *O*-trimethylsilyl pyrimidine ethers instantaneously hydrolyse to pyrimidin-2,4-dione **3**. Thus, reaction of silylated **3** with MOMCl afforded *N,N*-1,3-diMOM, *N*-1-MOM and *N*-3-MOM pyrimidine derivatives **4-6** in 22.4%, 13.4% and 11%, respectively (Scheme 1, method A). Furthermore, 1,1,1,3,3,3-hexamethyldisilazane (HMDS) in the presence of a catalytic amount of ammonium sulphate transformed uracil derivative **3** into the appropriate 2,4-bis(trimethylsilyloxy)pyrimidine, which was subsequently reacted with MOMCl to give *N,N*-1,3-diMOM and *N*-1-MOM compounds **4** and **5** in 2.9% and 21.4%, respectively (method B). 

In our search for a more efficient synthesis of compounds **4** and **5** an alternate method was applied using activated *N*-anionic uracil. Potassium carbonate was used as deprotonating agent and the thus *in situ* obtained uracil salt reacted with MOMCl in DMF to give *N,N*-1,3-diMOM, *N*-1-MOM and *N*-3-MOM pyrimidine derivatives **4-6** in 28.5%, 12.1% and 15.4% yields, respectively. The yields of **4** and **5** synthesized by the various methods are sumarized in Table 1. 

**Scheme 2:**
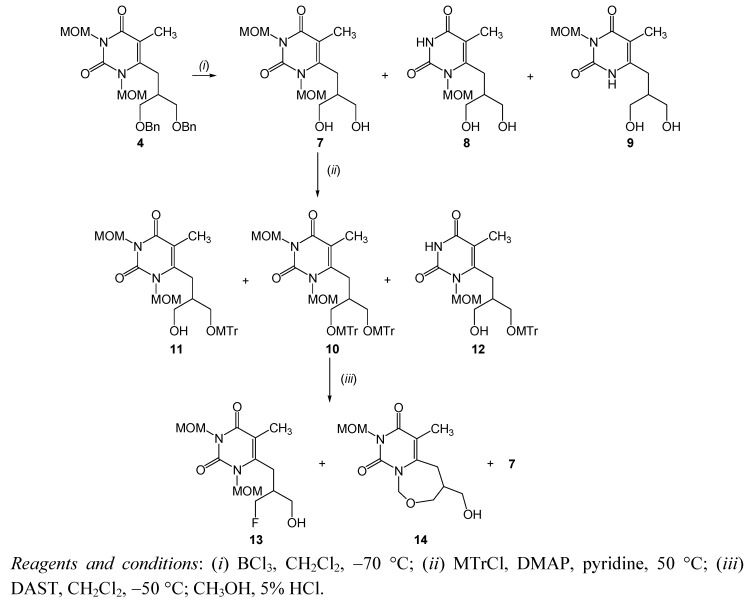
Syntheses of pyrimidine derivatives **7**-**14**.

Debenzylation of **4** was carried out using boron trichloride to afford *N,N*-1,3-diMOM (**7**) with 6-(1,3-dihydroxyisobutyl) side-chain as major product (33.4% yield). However, *N*-1 and *N*-3 deprotection of **4** also occurred during this reaction and *N*-1-MOM (**8**) and *N*-3-MOM (**9**) pyrimidine derivatives as a mixture of regioisomers in ratio 3:1 were isolated (Scheme 2). 

**Scheme 3:**
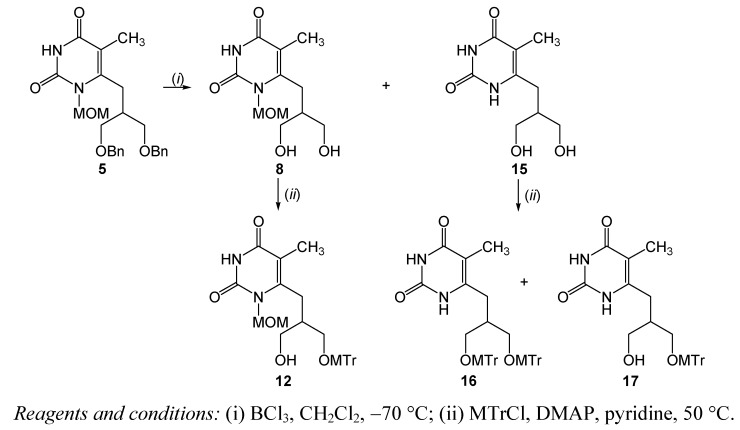
Syntheses of pyrimidine derivatives **8**, **12** and **15**-**17**.

*N,N*-1,3-diMOM-pyrimidine **7** was subsequently treated with 4-methoxytrityl chloride (MTrCl) to afford both ditritylated (compound **10**) and monotritylated (compounds **11** and **12**) pyrimidine derivatives. Fluorination of ditritylated compound **10** with diethylaminosulfur trifluoride (DAST) as the fluorinating reagent and subsequent *in situ* deprotection of trytl group by using HCl (5%) gave *N,N*-1,3-diMOM pyrimidines with 1-fluoro-3-hydroxyisobutyl- (compound **13**) and 1,3-dihydroxy-isobutyl (compound **7**) side-chains. The bicyclic compound **14**, was also obtained as an intramolecular cyclization product of **13** [45]. Interestingly, unexpected *N*-deprotection during debenzylation of **4** and tritylation of **7** occurred while in the detritylation reaction using HCl (5%) the MOM group was unreactive. The tosylation reaction of **11** and **12** did not afford the pyrimidine derivative with a 6-(1-methoxytrityl-3-tosylisobutyl) side-chain that could be used as the precursor for radiochemical synthesis. Debenzylation of **5** accompained by *N*-1 demethoxymethylation afforded both *N*-1-MOM pyrimidine derivative (**8**) and pyrimidin-2,4-dione (**15**) with a 6-(1,3-dihydroxyisobutyl) side-chain as the major product (Scheme 3). When the debenzylation of **5** was quenched after 2 h, instead of 4 h, only diol **8 **was obtained in a moderate 32% yield. Reaction of **8** with MTrCl gave monotritylated *N*-1-MOM pyrimidine derivative **12**. Similarly tritylation of **15** afforded both ditritylated (compound **16**) and monotritylated (compound **17**) pyrimidin-2,4-dione derivatives.

### 2.2. NMR Studies

**Figure 1:**
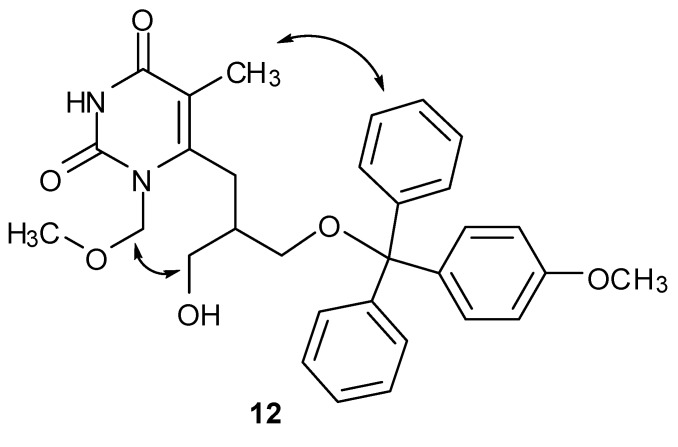
Predominant conformation of **12** as suggested by NOESY correlation signals. Key NOE interactions are indicated by double-headed arrows.

The chemical identities of compounds **2**−**17 **were confirmed by ^1^H-, ^13^C- and ^19^F-NMR measurements. Proton and fluorine NMR chemical shifts, as well as carbon NMR chemical shifts are reported in the Experimental section. *N*-1 and *N*-3 regioisomers were identified on the basis of heteronuclear ^1^H−^13^C correlation signals in 2D HMBC spectra and homonuclear ^1^H−^1^H correlations in NOESY spectra. NOESY cross-peaks demonstrate dipole–dipole interactions of nearby protons that are spatially within ca. 5 Å. Correlation signals were observed between methylene protons of MOM group (*δ* 5.19 ppm) and H-1’ (*δ* 2.75 ppm) as well as H-2’ (*δ* 2.20 ppm) in **5**, which suggested that MOM group is bound to *N*-1 of pyrimidine ring. Similarly, the methylene protons of the *N*-1-MOM group (*δ* 5.34 ppm) in **7** showed NOESY correlations with the H-1’ protons (*δ* 2.70 ppm), whereas the *N*-3-MOM group showed only NOE enhancements amongst the methoxymethyl protons. NOE cross-peaks between the phenyl ring (OMTr) and the CH_3_-5 group were observed in **12**, whereas the H-3” hydroxymethyl protons (*δ* 3.68 and 3.84 ppm) showed NOESY correlation signals with the *N*-1-MOM group (*δ* 5.34 ppm). These NOE enhancements suggested that predominant conformation of **12** possessed the hydroxymethyl group closer to the *N*-1-MOM functionality, whereas the OMTr was in proximity to the CH_3_-5 group (Figure 1). NOE enhancements were observed between the methylene protons of the *N*-1-MOM group (*δ* 5.28 ppm) and H-1’ (*δ* 2.72 ppm) as well as H-2’ (*δ* 2.10 ppm) in **13**. No NOE enhancements were observed between the *N*-1-MOM group and the hydroxymethyl or fluoromethyl protons, which suggests a nonrestricted rotation along the C-6 acyclic side-chain in **13**. Conformational study of compounds **5**, **6**, **7**, **10** and **16**, which bear identical substituents attached to C2', showed only trivial NOESY cross-peaks. Consequently, no particular conformational preferences could be established for these compounds.

## 3. Experimental

### 3.1. General

All solvents were dried/purified following recommended drying agents and/or distilled over 3 Å molecular sieves. For monitoring the progress of a reaction and for comparison purposes, thin layer chromatography (TLC) was performed on pre-coated *Merck* silica gel 60F-254 plates using an appropriate solvent system and the spots were detected under UV light (254 nm). For column chromatography silica gel (*Fluka*, 0.063-0.2 mm) was employed, glass column was slurry-packed under gravity and eluents were CH_2_Cl_2_/MeOH mixtures. Melting points (uncorrected) were determined with *Kofler* micro hot-stage (*Reichert*, Wien). ^1^H-, ^13^C- and ^19^F-NMR spectra were acquired on a *Bruker 300* MHz and Varian Unity Inova 300 MHz NMR spectrometers. All data were recorded in DMSO-*d*_6_ at 298 K. Chemical shifts were referenced to the residual solvent signal of DMSO at *δ* 2.50 ppm for ^1^H and *δ* 39.50 ppm for ^13^C. ^19^F-NMR chemical shifts were referenced externally with respect to CCl_3_F (*δ* 0.0 ppm). Individual resonances were assigned on the basis of their chemical shifts, signal intensities, multiplicity of resonances and H−H coupling constants. NOESY spectra were acquired with mixing time of 150 ms. Mass spectra were recorded on an *Agilent 6410* instrument equipped with electrospray interface and triple quadrupole analyzer (LC/MS/MS). High performance liquid chromatography was performed on an *Agilent 1100* series system with UV detection (photodiode array detector) using Zorbax C18 reverse-phase analytical column (2.1 × 30 mm, 3.5 µm). *6-[(3-Benzyloxy-2-benzyloxymethyl-2-hydroxy)propyl]-5-methyl-2,4-dimethoxypyrimidine* (**1**) and 6*-[(3-benzyloxy-2-benzyloxymethyl)propyl]-5-methyl-2,4-dimethoxypyrimidine* (**2**) were synthesized using analogous procedures as described previously [35,38]. 

### 3.2. Procedures for the Preparation of Compounds

#### *3.2.1. 6-[(3-Benzyloxy-2-benzyloxymethyl)propyl]-5-methylpyrimidin-2,4-dione* (**3**)

Method A: A solution of dry **2** (106 mg, 0.251 mmol), TMSCl (0.11 mL, 0.879 mmol) and NaI (130 mg, 0.879 mmol) in anhydrous CH_3_CN (5.5 mL) was stirred at r.t. for 20 h under Ar atmosphere. Solvent was evaporated *in vacuo* and residue was purified by column chromatography (CH_2_Cl_2_-MeOH = 20:1) to give pure oily **3** (18.1 mg, 18.3%).

Method B: Solution of **2** (488 mg, 1.15 mmol) in AcCl (17 mL) was refluxed for 20 h after that H_2_O (5 mL) was added and stirring was continued at r.t. overnight. Solvent was evaporated under reduced pressure and residue was chromatographed using silica column (CH_2_Cl_2_-MeOH = 20:1) to afford oily product **3** (308.7 mg, 67.8%). 

Analytical data: ^1^H-NMR: 10.92 (1H, s, NH-3), 10.53 (1H, s, NH-1), 7.25−7.35 (10H, m, Ph), 4.44 (4H, s, H-4',4''), 3.43 (4H, d, *J* = 5.16, H-3',3''), 2.42 (2H, d, *J* = 7.11, H-1'), 2.27 (1H, m, H-2'), 1.71 (3H, s, CH_3_-5). ^13^C-NMR: 164.74 (C-4), 150.82 (C-2), 149.18 (C-6), 138.43 (C-Ph_quat_), 127.30−128.15 (CH-Ph), 105.16 (C-5), 72.07 (C-4',4''), 69.34 (C-3',3''), 37.95 (C-2'), 29.48 (C-1'), 9.61 (CH_3_-5). Positive ESI-MS 395 (M+H). Anal. Calcd for C_23_H_26_N_2_O_4_: C 70.03, H 6.64. Found: C 69.96, H 6.67.

#### 3.2.2. *6-[(3-Benzyloxy-2-benzyloxymethyl)propyl]-1,3-dimethoxymethyl-5-methylpyrimidin-2,4-dione* (**4**), *6-[(3-benzyloxy-2-benzyloxymethyl)propyl]-1-methoxymethyl-5-methylpyrimidin-2,4-dione* (**5**) and *6-[(3-benzyloxy-2-benzyloxymethyl)propyl]-3-methoxymethyl-5-methylpyrimidin-2,4-dione* (**6**)

Method A: To a solution of dry **3** (33.7 mg, 0.085 mmol) in anhydrous CH_3_CN (2 mL) TMSCl (0.03 mL, 0.256 mmol) was added and reaction mixture was refluxed for 2 h. Then reaction mixture was cooled to r.t. and MOMCl (0.017 mL, 0.214 mmol) and (*i*Pr)_2_EtN (0.037 mL, 0.214 mmol) were added. Stirring at 50 °C was continued for 6 h. Solvent was evaporated to dryness and residue was purified by column chromatography (CH_2_Cl_2_-MeOH = 30:1) to give oily products **4** (9.2 mg, 22.4%), **5** (5 mg, 13.4%) and **6** (4.1 mg, 11%). 

Method B: A solution of dry **3** (200 mg, 0.507 mmol) and (NH_4_)_2_SO_4_ (13.4 mg, 0.1 mmol) in HMDS (3 mL, 14.4 mmol) was refluxed for 1 h under Ar atmosphere. Reaction mixture was cooled to r.t. and MOMCl (0.12 mL, 1.521 mmol) was added. Stirring was continued at r.t. overnight and solvent was evaporated. The residue was purified by column chromatography (CH_2_Cl_2_-MeOH = 30:1) to afford **4** (7.1 mg, 2.9%) and **5** (47.5 mg, 21.4%).

Method C: To a solution of dry **3** (644.8 mg, 1.635 mmol) and K_2_CO_3_ (1.13 g, 8.177 mmol) in anhydrous DMF (6 mL) at −15 °C MOMCl (0.5 mL, 6.54 mmol) was added. Reaction mixture was stirred at r.t. overnight. Solvent was evaporated *in vacuo* and residue was purified by column chromatography (CH_2_Cl_2_-MeOH = 30:1) to yield **4** (224.8 mg, 28.5%), **5** (86.8 mg, 12.1%) and **6** (110.2 mg, 15.4%).

*6-[(3-Benzyloxy-2-benzyloxymethyl)propyl]-1,3-dimethoxymethyl-5-methyl-pyrimidin-2,4-dione* (**4**). ^1^H-NMR: 7.29−7.32 (10H, m, Ph), 5.25 (2H, s, CH_2_-N1), 5.20 (2H, s, CH_2_-N3), 4.46 (4H, s, H-4',4''), 3.38 (4H, m, H-3',3''), 3.26 (3H, s, OCH_3_), 3.21 (3H, s, OCH_3_), 2.78 (2H, d, *J* = 6.72 Hz, H-1'), 2.27 (1H, m, H-2'), 1.87 (3H, s, CH_3_-5). ^13^C-NMR: 165.74 (C-4), 152.35 (C-2), 150.08 (C-6), 138.69 (C-Ph_quat_), 127.89−128.71 (CH-Ph), 108.86 (C-5), 74.81 (CH_2_-N1), 73.99 (CH_2_-N3), 72.97 (C-4',4''), 70.10 (C-3',3''), 57.53 (OCH_3_-N1), 56.38 (OCH_3_-N3), 38.33 (C-2'), 30.25 (C-1'), 12.05 (CH_3_-5). Positive ESI-MS 483 (M+H). Anal. Calcd for C_27_H_34_N_2_O_6_: C 67.20, H 7.10. Found: C 67.26, H 7.07.

*6-[(3-Benzyloxy-2-benzyloxymethyl)propyl]-1-methoxymethyl-5-methylpyrimidin-2,4-dione* (**5**). ^1^H- NMR: 11.34 (1H, s, NH-3), 7.2−7.4 (10H, m, Ph), 5.19 (2H, s, CH_2_-N1), 4.46 (4H, s, H-4',4''), 3.45 (4H, m, H-3',3''), 3.19 (3H, s, OCH_3_), 2.75 (2H, d, *J* = 7.1 Hz, H-1'), 2.20 (1H, m, H-2'), 1.80 (3H, s, CH_3_-5). ^13^C-NMR: 164.07 (C-4), 152.04 (C-2), 150.31 (C-6), 138.86 (C-Ph_quat_), 127.91−128.71 (CH-Ph), 109.52 (C-5), 73.73 (CH_2_-N1), 72.67 (C-4',4''), 70.05 (C-3',3''), 57.22 (OCH_3_), 39.47 (C-2'), 29.47 (C-1'), 11.41 (CH_3_-5). Positive ESI-MS 439 (M+H). Anal. Calcd for C_25_H_30_N_2_O_5_: C 68.47, H 6.90. Found: C 68.54, H 6.88.

*6-[(3-Benzyloxy-2-benzyloxymethyl)propyl]-3-methoxymethyl-5-methylpyrimidin-2,4-dione* (**6**). ^1^H- NMR: 10.88 (1H, s, NH-1), 7.2−7.4 (10H, m, Ph), 5.13 (2H, s, CH_2_-N3), 4.44 (4H, s, H-4',4''), 3.44 (4H, d, *J* = 5.4 Hz, H-3',3''), 3.24 (3H, s, OCH_3_), 2.46 (2H, d, *J* = 7.6 Hz, H-1'), 2.29 (1H, m, H-2'), 1.77 (3H, s, CH_3_-5). ^13^C-NMR: 163.69 (C-4), 151.38 (C-2), 149.35 (C-6), 138.72 (C-Ph_quat_), 127.91−128.72 (CH-Ph), 109.17 (C-5), 72.54 (CH_2_-N3), 69.84 (C-4',4''), 68.88 (C-3',3''), 56.20 (OCH_3_), 38.58 (C-2'), 27.54 (C-1'), 10.79 (CH_3_-5). Positive ESI-MS 439 (M+H). Anal. Calcd for C_25_H_30_N_2_O_5_: C 68.47, H 6.90. Found: C 68.41, H 6.93.

#### 3.2.3. *6-[(3-Hydroxy-2-hydroxymethyl)propyl]-1,3-dimethoxymethyl-5-methylpyrimidin-2,4-dione* (**7**), *6-[(3-hydroxy-2-hydroxymethyl)propyl]-1-methoxymethyl-5-methylpyrimidin-2,4-dione* (**8**) and *6-[(3-hydroxy-2-hydroxymethyl)propyl]-3-methoxymethyl-5-methylpyrimidin-2,4-dione* (**9**)

A solution of dry **4** (122.3 mg, 0.253 mmol) in anhydrous CH_2_Cl_2_ (6.4 mL) was cooled to −78 °C with exclusion of moisture and BCl_3_ (1 mL, 1M in CH_2_Cl_2_) was added under Ar atmosphere. The reaction mixture was stirred at −70 °C for 1 h and quenched by the addition of CH_2_Cl_2_/MeOH solution (1:1, 10 mL) and evaporated to dryness. Purification by column chromatography (CH_2_Cl_2_-MeOH = 10:1) gave **7** as an oil (25.7 mg, 33.4%) and **8 **and **9** as a mixture of *N*-1 and *N*-3 regioisomers in a 3:1 ratio (16.2 mg, 24.8%).

*6-[(3-Hydroxy-2-hydroxymethyl)propyl]-1,3-dimethoxymethyl-5-methylpyrimidin-2,4-dione* (**7**). ^1^H-NMR: 5.34 (2H, s, CH_2_-N1), 5.21 (2H, s, CH_2_-N3), 4.67 (2H, t, *J* = 4.9 Hz, OH), 3.40 (4H, m, H-3',3''), 3.27 (6H, s, 2 × OCH_3_), 2.70 (2H, d, *J* = 7.1 Hz, H-1'), 1.90 (3H, s, CH_3_-5), 1.79 (1H, m, H-2'). ^13^C NMR: 164.14 (C-4), 152.45 (C-2), 151.03 (C-6), 108.59 (C-5), 74.65 (CH_2_-N1), 72.43 (CH_2_-N3), 61.24 (C-3',3''), 57.40 (OCH_3_-N1), 56.40 (OCH_3_-N3), 43.91 (C-2'), 27.03 (C-1'), 12.15 (CH_3_-5). Positive ESI-MS 303 (M+H). Anal. Calcd for C_13_H_22_N_2_O_6_: C 51.65, H 7.33. Found: C 51.70, H 7.34.

*6-[(3-Hydroxy-2-hydroxymethyl)propyl]-1-methoxymethyl-5-methylpyrimidin-2,4-dione* (**8**). ^1^H-NMR: 11.28 (1H, s, NH-3), 5.27 (2H, s, CH_2_-N1), 4.62 (2H, t, *J* = 4.89 Hz, OH), 3.38 (4H, m, H-3',3''), 3.17 (3H, s, OCH_3_), 2.67 (2H, d, *J* = 6.9 Hz, H-1'), 2.46 (1H, m, H-2'),1.84 (3H, s, CH_3_-5). ^13^C-NMR: 163.74 (C-4), 152.13 (C-2), 151.38 (C-6), 109.21 (C-5), 73.59 (CH_2_-N1), 61.24 (C-3',3''), 56.23 (OCH_3_), 43.93 (C-2'), 26.93 (C-1'), 11.50 (CH_3_-5).

*6-[(3-Hydroxy-2-hydroxymethyl)propyl]-3-methoxymethyl-5-methylpyrimidin-2,4-dione* (**9**). ^1^H-NMR: 10.78 (1H, s, NH-1), 5.15 (2H, s, CH_2_-N3), 4.51 (2H, t, *J* = 5.06 Hz, OH), 3.26 (3H, s, OCH_3_), 2.37 (2H, d, *J* = 6.9 Hz, H-1'), 2.27 (1H, m, H-2'), 1.80 (3H, s, CH_3_-5). ^13^C-NMR: 163.10 (C-4), 151.43 (C-2), 150.96 (C-6), 109.0 (C-5), 71.26 (CH_2_-N3), 61.13 (C-3',3''), 56.84 (OCH_3_), 43.10 (C-2'), 26.41 (C-1'), 10.82 (CH_3_-5).

#### 3.2.4. *6-[2,3-Bis(4-methoxytriphenylmethoxymethyl)propyl]-1,3-dimethoxymethyl-5-methylpyrimidin-2,4-dione* (**10**), *6-[3-hydroxy-2-(4-methoxytriphenylmethoxymethyl)propyl]-1,3-dimethoxymethyl-5-methylpyrimidin-2,4-dione* (**11**) and *6-[3-hydroxy-2-(4-methoxytriphenylmethoxymethyl)propyl]-1-methoxymethyl-5-methylpyrimidin-2,4-dione* (**12**)

A solution of **7** (23.7 mg, 0.078 mmol) and DMAP (0.2 mg, 0.0016 mmol) in DMF (0.5 mL) and Et_3_N (0.04 mL) was cooled to 0 °C and after 10 minutes MTrCl (60.52 mg, 0.196 mmol) was added. Obtained mixture was additionally stirred at r.t. overnight and solvent was evaporated. Further purification by column chromatography (initial eluent CH_2_Cl_2_-CH_3_OH = 50:1, then CH_2_Cl_2_-CH_3_OH = 10:1) afforded gray crystals of **10** (32 mg, 46.4%, m.p. = 101–102 °C), yellow powder of **11** (16.9 mg, 37.5%, m.p. = 57–59 °C) and white powder of **12** (2.3 mg, 5.5%, m.p. = 94–96 °C).

*6-[2,3-Bis(4-methoxytriphenylmethoxymethyl)propyl]-1,3-dimethoxymethyl-5-methylpyrimidin-2,4-dione* (**10**). ^1^H-NMR: 7.2−7.4 (20H, m, Ph), 7.14 (4H, m, Ph), 6.8 (4H, m, Ph), 5.17 (2H, s, CH_2_-N1), 5.17 (2H, s, CH_2_-N3), 3.73 (6H, s, OCH_3_), 3.20 (6H, s, OCH_3_), 3.19 (2H, m, H-3''), 3.00 (2H, m, H-3'), 2.67 (2H, m, H-1'), 2.22 (1H, m, H-2'), 1.60 (3H, s, CH_3_-5). ^13^C-NMR: 162.43 (C-4), 158.63 (C-Ph_quat_-12'), 152.25 (C-2), 149.74 (C-6), 144.63 (C-Ph_quat_-5',5''), 135.35 (C-Ph_quat_-9'), 130.37 (CH-Ph-10',10''), 128.28−130.38 (CH-Ph-6'-7''), 127.31 (CH-Ph-8',8''), 113.57 (CH-Ph-11',11''), 108.75 (C-5), 86.49 (C-4',4''), 73.75 (CH_2_-N1), 72.32 (CH_2_-N3), 63.96 (C-3',3''), 57.32 (OCH_3_-N1), 56.70 (OCH_3_-N3), 55.79 (OCH_3_-MTr), 38.65 (C-2'), 30.60 (C-1'), 12.22 (CH_3_-5). Positive ESI-MS 847 (M+H). Anal. Calcd for C_53_H_54_N_2_O_8_: C 75.16, H 6.43. Found: C 75.04 , H 6.46.

*6-[3-Hydroxy-2-(4-methoxytriphenylmethoxymethyl)propyl]-1,3-dimethoxymethyl-5-methylpyrimidin-2,4-dione* (**11**).^ 1^H-NMR: 7.2−7.4 (10H, m, Ph), 7.18 (2H, m, Ph), 6.87 (2H, m, Ph), 5.24 (2H, s, CH_2_-N1, 5.20 (1H, m, CH_2_-N3), 5.18 (1H, m, CH_2_-N3), 4.77 (1H, t, *J* = 4.5 Hz, OH), 3.74 (3H, s, OCH_3_), 3.46 (2H, m, H-3''-OH), 3.24 (3H, s, OCH_3_), 3.23 (3H, s, OCH_3_), 2.92 (2H, m, H-3'-OMTr), 2.69 (2H, m, H-1'), 2.03 (1H, m, H-2'), 1.74 (3H, s, CH_3_-5). ^13^C-NMR: 162.59 (C-4), 158.61 (C-Ph_quat_-12'), 152.36 (C-2), 150.46 (C-6), 144.87, 144.68 (C-Ph_quat_-5',5''), 135.49 (C-Ph_quat_-9'), 130.37 (CH-Ph-10',10''), 128.27−128.48 (CH-Ph-6'-7''), 127.30 (CH-Ph-8',8''), 113.59 (CH-Ph-11',11''), 108.65 (C-5), 86.50 (C-4',4''), 73.71 (CH_2_-N1), 72.14 (CH_2_-N3), 64.24, 61.60 (C-3',3''), 57.39 (OCH_3_-N1), 56.56 (OCH_3_-N3), 55.49 (OCH_3_-MTr), 42.03 (C-2'), 27.98 (C-1'), 12.18 (CH_3_-5). Positive ESI-MS 575 (M+H). Anal. Calcd for C_33_H_38_N_2_O_7_: C 68.97, H 6.67. Found: C 69.05, H 6.70.

*6-[3-Hydroxy-2-(4-methoxytriphenylmethoxymethyl)propyl]-1-methoxymethyl-5-methylpyrimidin-2,4-dione* (**12**).^ 1^H-NMR: 11.22 (1H, s, NH-3), 7.2−7.4 (10H, m, Ph), 7.20 (2H, m, Ph), 6.87 (2H, m, Ph), 5.34 (2H, s, CH_2_-N1), 3.84 (1H, m, H-3''-OH), 3.74 (3H, s, OCH_3_), 3.68 (1H, m, H-3''-OH), 3.22 (3H, s, OCH_3_), 3.03 (2H, m, H-3'-OMTr), 2.73 (2H, m, H-1'), 2.18 (1H, m, H-2'), 1.74 (3H, s, CH_3_-5). ^13^C- NMR: 165.08 (C-4), 158.64 (C-Ph_quat_-12'), 150.83 (C-2), 149.20 (C-6), 144.92 (C-Ph_quat_-5',5''), 135.40 (C-Ph_quat_-9'), 130.36 (CH-Ph-10',10''), 128.30−128.36 (CH-Ph-6'-7''), 127.32 (CH-Ph-8',8''), 113.64 (CH-Ph-11',11''), 109.47 (C-5), 86.44 (C-4'), 73.77 (CH_2_-N1), 68.68 (C-3',3''), 59.52 (OCH_3_-N1), 55.49 (OCH_3_-MTr), 44.05 (C-2'), 28.85 (C-1'), 11.17 (CH_3_-5). Positive ESI-MS 531 (M+H). Anal. Calcd for C_31_H_34_N_2_O_6_: C 70.17, H 6.46. Found: C 70.25, H 6.44.

#### 3.2.5. *6-[(3-Fluoro-2-hydroxymethyl)propyl]-1,3-dimethoxymethyl-5-methylpyrimidin-2,4-dione* (**13**), *6-[(3-hydroxy-2-hydroxymethyl)propyl]-1,3-dimethoxymethyl-5-methylpyrimidin-2,4-dione* (**7**) and *4-hydroxymethyl-8-methoxymethyl-6-methyl-4,5-dihydro-pyrimido[1,6-c][1,3]oxazepine-7,9-dione* (**14**)

A solution of dry **10** (30 mg, 0.037 mmol) in anhydrous CH_2_Cl_2_ (8 mL) was cooled to −78 °C and stirred for 15 min under Ar atmosphere. DAST (0.05 mL) was added dropwise and reaction was kept at −78 °C for additional 15 min after which cooling bath was removed. After 5 h of stirring at r.t., saturated aqueous solution of NaHCO_3_ (10 mL) was added and reaction was partitioned. Organic layer was separated, dried over MgSO_4_ and evaporated to dryness. Raw product was then dissolved in CH_3_OH (0.5 mL) and 5% HCl (0.7 mL) and refluxed for 15 min. Solvent was evaporated and residue was purified by column chromatography (CH_2_Cl_2_-CH_3_OH = 30:1) to afford **13** (1.2 mg, 10.7%), **7** (3.6 mg, 32.2%) and **14** (3.1 mg, 31%).

*6-[(3-Fluoro-2-hydroxymethyl)propyl]-1,3-dimethoxymethyl-5-methylpyrimidin-2,4-dione* (**13**).^ 1^H- NMR: 5.28 (2H, s, CH_2_-N1), 5.22 (2H, s, CH_2_-N3), 4.92 (1H, t, *J* = 4.9 Hz, OH), 4.45 (2H, ddd, *J* = 47.6, 4.9, 1.9 Hz, CH_2_F-3''), 3.45 (2H, m, H-3'-OH), 3.28 (6H, s, OCH_3_), 2.72 (2H, m, H-1'), 2.10 (1H, m, H-2'), 1.89 (3H, s, CH_3_-5). ^19^F-NMR: 225.79 (td, *J* = 47.9, 24.8 Hz). ^13^C-NMR: 162.73 (C-4), 152.45 (C-2), 149.63 (C-6), 109.06 (C-5), 83.67 (d, *J* = 165.29, CH_2_F-3''), 74.88 (CH_2_-N1), 72.50 (CH_2_-N3), 60.05 (d, *J* = 5.94 Hz, C-3'), 57.42 (OCH_3_-N1), 56.47 (OCH_3_-N3), 41.66 (d, *J* = 17.74 Hz, C-2'), 26.32 (d, *J* = 4.96 Hz, C-1'), 12.10 (CH_3_-5). Positive ESI-MS 305 (M+H). Anal. Calcd for C_13_H_21_FN_2_O_5_: C 51.31, H 6.96. Found: C 51.27, H 6.98.

*4-Hydroxymethyl-8-methoxymethyl-6-methyl-4,5-dihydro-pyrimido[1,6-c][1,3]oxazepine-7,9-dione* (**14**). ^1^H-NMR: 5.58 (1H, d, *J* = 12.0 Hz, H-4'), 5.38 (1H, d, *J* = 12.0 Hz, H-4'), 5.20 (2H, s, CH_2_-N3), 3.87 (1H, dd, *J* = 11.8, 3.5 Hz, H-3''), 3.68 (1H, dd, *J* = 11.8, 7.4 Hz, H-3''), 3.31 (2H, m, H-3'-OH), 3.26 (3H, s, OCH_3_), 3.06 (1H, d, *J* = 15.1 Hz, H-1'), 2.92 (1H, dd, *J* = 15.1, 9.1 Hz, H-1'), 1.93 (3H, s, CH_3_-5), 1.91 (1H, m, H-2'). ^13^C-NMR: 162.92 (C-4), 151.02 (C-2), 149.51 (C-6), 106.87 (C-5), 73.52 (CH_2_-N1), 72.43 (CH_2_-N3), 61.45 (CH_2_-O), 57.38 (CH_2_-OH), 56.32 (OCH_3_), 33.77 (CH), 30.59 (C-1'), 11.33 (CH_3_-5). Positive ESI-MS 271 (M+H). Anal. Calcd for C_12_H_18_N_2_O_5_: C 53.33, H 6.71. Found: C 53.29, H 6.68.

#### 3.2.6. *6-[(3-Hydroxy-2-hydroxymethyl)propyl]-1-methoxymethyl-5-methylpyrimidin-2,4-dione* (**8**) and *6-[(3-hydroxy-2-hydroxymethyl)propyl]-5-methylpyrimidin-2,4-dione* (**15**)

Method A: A solution of compound **5** (43.7 mg, 0.1 mmol) in anhydrous CH_2_Cl_2_ (2.3 mL) was cooled to −78 °C with exclusion of moisture and BCl_3_ (0.4 mL, 1M in CH_2_Cl_2_) was added under Ar atmosphere. The reaction mixture was stirred at −70 °C for 4 h after that was quenched by the addition of CH_2_Cl_2_-MeOH solution (1:1, 3 mL) and evaporated to dryness. After column chromatography (CH_2_Cl_2_-MeOH = 10:1) compound **8** as an oil (4.8 mg, 18.6%) and **15** as a white crystals (12.8 mg, 60%, m.p. = 117–119 °C) were isolated.

Method B: A solution of compound **5** (41.1 mg, 0.09 mmol) in anhydrous CH_2_Cl_2_ (2.2 mL) was cooled to −78 °C with exclusion of moisture and BCl_3_ (0.38 mL, 1M in CH_2_Cl_2_) was added under Ar atmosphere. The reaction mixture was stirred at −70 °C for 2 h after that was quenched by the addition of CH_2_Cl_2_-MeOH solution (1:1, 3 mL) and evaporated to dryness. After column chromatography (CH_2_Cl_2_-MeOH = 10:1) compound **8** as an oil (7.7 mg, 31.7%) was isolated.

*6-[(3-Hydroxy-2-hydroxymethyl)propyl]-5-methylpyrimidin-2,4-dione* (**15**).^ 1^H-NMR: 11.03 (1H, s, NH-3), 10.54 (1H, s, NH-1), 4.64 (2H, bs, OH), 3.53 (4H, m, H-3',3''), 2.75 (2H, d, *J* = 7.2 Hz, H-1'), 1.98 (1H, m, H-2'), 1.89 (3H, s, CH_3_-5). ^13^C-NMR: 164.80 (C-4), 150.76 (C-2), 150.14 (C-6), 104.79 (C-5), 60.71 (C-3',3''), 42.55 (C-2'), 29.02 (C-1'), 9.62 (CH_3_-5). Positive ESI-MS 215 (M+H). Anal. Calcd for C_9_H_14_N_2_O_4_: C 50.46, H 6.59. Found: C 50.51, H 6.61.

#### 3.2.7. *6-[3-Hydroxy-2-(4-methoxytriphenylmethoxymethyl)propyl]-1-methoxymethyl-5-methyl-pyrimidin-2,4-dione* (**12**)

A solution of **8** (5.2 mg, 0.02 mmol) and DMAP (0.05 mg, 0.43 µmol) in DMF (0.5 mL) and Et_3_N (0.01 mL) was cooled to 0 °C and after 10 minutes MTrCl (16.8 mg, 0.054 mmol) was added. The obtained mixture was additionally stirred at r.t. overnight and the solvent was evaporated. Further purification by column chromatography (initial eluent CH_2_Cl_2_-CH_3_OH = 50:1, then CH_2_Cl_2_-CH_3_OH = 10:1) afforded **12** as a white powder (3.3 mg, 30.8%, m.p. = 94–96 °C).

#### 3.2.8. *6-[2,3-Bis(4-methoxytriphenylmethoxymethyl)propyl]-5-methylpyrimidin-2,4-dione* (**16**) and *6-[3-hydroxy-2-(4-methoxytriphenylmethoxymethyl)propyl]-5-methylpyrimidin-2,4-dione* (**17**)

A solution of **15** (9.8 mg, 0.046 mmol) and DMAP (0.12 mg, 0.001 mmol) in DMF (0.5 mL) and Et_3_N (0.02 mL) was cooled to 0 °C and after 10 min MTrCl (34.34 mg, 0.114 mmol) was added. The obtained mixture was additionally stirred at room temperature for 2 h and the solvent was then evaporated. Further purification by column chromatography (initial eluent CH_2_Cl_2_-CH_3_OH = 50:1, then CH_2_Cl_2_-CH_3_OH = 10:1) afforded a yellow powder of **16** (5.9 mg, 26.12%, m.p. = 136–138 °C) and white crystals of **17** (2.1 mg, 9.4%, m.p. = 104–106 °C).

*6-[2,3-Bis(4-methoxytriphenylmethoxymethyl)propyl]-5-methylpyrimidin-2,4-dione* (**16**).^ 1^H-NMR: 10.84 (1H, s, NH-3), 10.48 (1H, s, NH-1), 7.2−7.4 (20H, m, Ph), 7.15 (4H, m, Ph), 6.83 (4H, m, Ph), 3.73 (6H, s, OCH_3_), 3.12 (2H, m, H-3'), 2.99 (2H, m, H-3''), 2.52 (2H, m, H-1'), 2.31 (1H, m, H-2'), 1.50 (3H, s, CH_3_-5). ^13^C-NMR: 165.07 (C-4), 158.57 (CH-Ph-12'), 151.24 (C-2), 149.47 (C-6), 144.86 (CH-Ph-5',5''), 135.54 (CH-Ph-9'), 130.37 (CH-Ph-10',10''), 128.21−130.32 (CH-Ph-6'-7''), 127.24 (CH-Ph-8',8''), 113.52 (CH-Ph-11',11''), 105.35 (C-5), 86.20 (C-4',4''), 63.63 (C-3',3''), 55.48 (OCH_3_), 30.77 (C-1'), 30.06 (C-2'), 10.15 (CH_3_-5). Positive ESI-MS 759 (M+H). Anal. Calcd for C_49_H_46_N_2_O_6_: C 77.55, H 6.11. Found: C 77.64, H 6.07.

*6-[3-Hydroxy-2-(4-methoxytriphenylmethoxymethyl)propyl]-5-methylpyrimidin-2,4-dione* (**17**).^ 1^H- NMR: 10.85 (1H, s, NH-3), 10.43 (1H, s, NH-1), 7.22−7.34 (10H, m, Ph), 7.17 (2H, d, *J* = 8.97 Hz, Ph), 6.85 (2H, d, *J* = 8.85 Hz, Ph), 4.55 (1H, t, *J* = 4.67 Hz, OH), 3.72 (3H, s, OCH_3_), 3.42 (2H, t, *J* = 4.85 Hz, H-3'), 3.36 (2H, m, H-3''), 2.97 (2H, m, H-1'), 2.32 (1H, m, H-2'), 1.59 (3H, s, CH_3_-5). ^13^C NMR: ^13^C-NMR: 164-98 (C-4), 158.51 (CH-Ph-12'), 150.78 (C-2), 149.52 (C-6), 144.85 (CH-Ph-5',5''), 135.57 (CH-Ph-9'), 130.34 (CH-Ph-10',10''), 128.22− 130.36 (CH-Ph-6'-7''), 127.27 (CH-Ph-8',8''), 113.48 (CH-Ph-11',11''), 106.81 (C-5), 85.73 (C-4'), 64.09, 63.65 (C-3',3''), 55.39 (OCH_3_), 31.14 (C-2'), 27.07 (C-1'), 10.99 (CH_3_-5). Positive ESI-MS 487 (M+H). Anal. Calcd for C_29_H_30_N_2_O_5_: C 71.59, H 6.21. Found: C 71.51, H 6.24.

## 4. Conclusions

In summary, we have adopted simple and efficient methods for the protection and deprotection of the carbonyl and nitrogen moieties in a pyrimidine ring, as well as hydroxyl groups in a C-6 isobutyl side-chain under mild conditions in moderate to excellent yields. The methoxymethyl (MOM) moiety as protecting group was introduced using different synthetic methods. Two methods performed by silylation of uracil and *in situ* reaction of *O*-persilylated uracil with MOMCl gave *N*-1- and/or *N*-3-MOM pyrimidine derivatives **4**−**6**. A synthetic approach using activated an *N*-anionic pyrimidine derivative afforded desired *N,N*-1,3-diMOM and *N*-1-MOM pyrimidines **4** and **5** in good yield. *N*-1 and *N*-3 regioisomers were assigned on the basis of heteronuclear ^1^H−^13^C correlation signals in 2D HMBC spectra and homonuclear ^1^H−^1^H correlations in NOESY spectra. Thus, NOE interactions between the methylene protons of a MOM group and H-1’ as well as H-2’ in **5** revealed that the MOM group is bound to *N*-1 of the pyrimidine ring. The removal of benzyl protecting groups in **4 **and **5** was accomplished using boron trichloride to give 6-(1,3-dihydroxyisobutyl)-*N*-MOM pyrimidines **7 **and **8** as a major products. Pyrimidine derivatives **7**, **8 **and **15** with free hydroxyl functionalities were subsequently converted to ditritylated (compounds **10** and **16**) and monotritylated (compounds **11**, **12** and **17**) derivatives. It is interesting to note that debenzylation of **4** and **5** and tritylation of **7** was accompanied with removal of the *N*-MOM protecting group. For preparation of precursor for ^18^F radiolabelling that contains appropriate leaving groups, introduction of mesylate, instead of tosylate, as less bulky group is foreseen. 
